# Complex Regional Pain Syndrome: Is There a Role for Capsaicin?

**DOI:** 10.7759/cureus.34179

**Published:** 2023-01-25

**Authors:** Ana Raquel S Cruz, Filipa R Sales, Filipa Maldonado, Joana Torres

**Affiliations:** 1 Anesthesiology, Hospital Pedro Hispano, Matosinhos, PRT

**Keywords:** pain medicine, trauma, chronic pain, capsaicin, complex regional pain syndrome

## Abstract

Complex regional pain syndrome (CRPS) is a challenging disorder occurring in patients most often after trauma or surgery. Its treatment is very complex, and even then, no treatment is fully effective. Capsaicin is a well-accepted treatment for neuropathic pain. However, its use in CRPS is controversial, with few studies having been published on it. In this case report, we describe the case of a female patient with CPRS type II, whose treatment with topical capsaicin resulted in great functional improvement. The patient was referred to the Pain Medicine Unit due to a CRPS type II due to trauma in her right wrist. She complained of severe pain in the median nerve territory of her dominant hand, associated with hyperalgesia, allodynia, burning, and electric shock sensation, resulting in functional disability. Electromyography was compatible with severe axonal injury of the right median nerve of the wrist. After conventional therapies were tried with no improvement, treatment with a capsaicin 8% patch was proposed. A functional improvement was observed after two applications of the capsaicin treatment, allowing the patient to regain activity in her hand. This shows that although evidence for capsaicin use in CRPS treatment is scarce, it can be a viable alternative for some patients.

## Introduction

Complex regional pain syndrome (CRPS) is a debilitating disorder occurring most often after trauma and surgery, being associated with sensory, motor, autonomic, skin, or bone changes. It is considered a multifactorial condition, with different mechanisms involved in it. However, an increased inflammatory response seems to be one of the most consistent findings. It is an exclusion diagnosis and requires the existence of pain, allodynia or hyperalgesia not proportional to injury, and evidence of dystrophic signs and symptoms. CRPS type II occurs when there is associated nerve injury [1,2].

The management of CRPS can be truly challenging, and there is no proven cure for it. Recognized therapies combine pharmacological, physical, and psychological therapies. However, some patients still suffer from persistent symptoms. Capsaicin is an approved and well-established treatment for neuropathic pain [3]. However, evidence for its use in CRPS, which is still off-label, is scarce.

In this case report, we describe the case of a patient with CPRS type II under treatment with topical capsaicin, with great functional improvement.

This article was previously presented as a poster at the 2021 Congress of the Portuguese Society of Anesthesiology.

## Case presentation

A female patient in her 40s, a house cleaner with a history of mild asthma, was a victim of a domestic accident that resulted in trauma in her right wrist. She was urgently submitted to flexor tenorrhaphy and median nerve neurorrhaphy. Two months later, a CRPS type II was suspected, and the patient was referred early to Pain Medicine Unit. 

**Figure 1:**
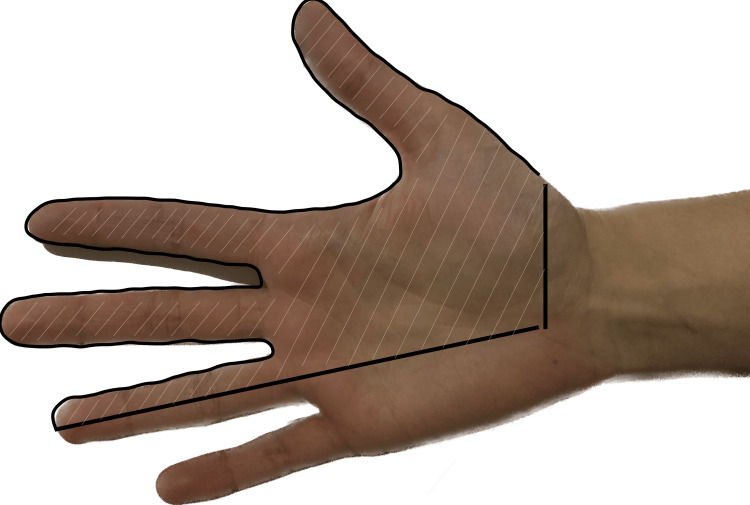
Painful area before treatment with capsaicin

In the initial evaluation, the patient complained of severe pain - visual analog scale (VAS) of 10 - in all the median nerve territory of her dominant hand (Figure 1), associated with hyperalgesia, allodynia, burning, and electric shock sensation.

Concomitantly, exuberant hand edema and flushing were observed. This clinical scenario resulted in functional disability, and she had to quit her job and struggled with relational problems, depression symptoms, and insomnia. She was followed up by a psychiatrist and medicated with antidepressants.

After the first evaluation in the Pain Medicine Unit, she started treatment with gabapentin 300 mg three times a day, amitriptyline 10 mg, and tramadol, and an electrophysiological study of the upper limbs was requested.

The electrophysiological study demonstrated an “absence of sensory potentials on the fingers of the right hand to stimulation of the right median nerve of the wrist, compatible with severe axonal injury.”

After one month, no improvement was observed, and the functional disability persisted. Due to refractoriness to first-line therapy, the patient was proposed for treatment with a capsaicin 8% patch along with previous medications. The patch was applied in the affected area, having already been drawn and adequately prepared by our nurse pain specialist. Capsaicin was applied in the Pain Medicine Unit for about 60 minutes uneventfully. 

One week later, the patient reported a great improvement, experiencing no pain. Three months after the first capsaicin 8% patch application, in the reevaluation consultation, the hyperalgesia and allodynia area, as well as the edema and flushing of the right hand, were found to have reduced considerably. Due to some persistent symptoms, gabapentin was increased to 400 mg three times a day, and a second patch was applied.

**Figure 2:**
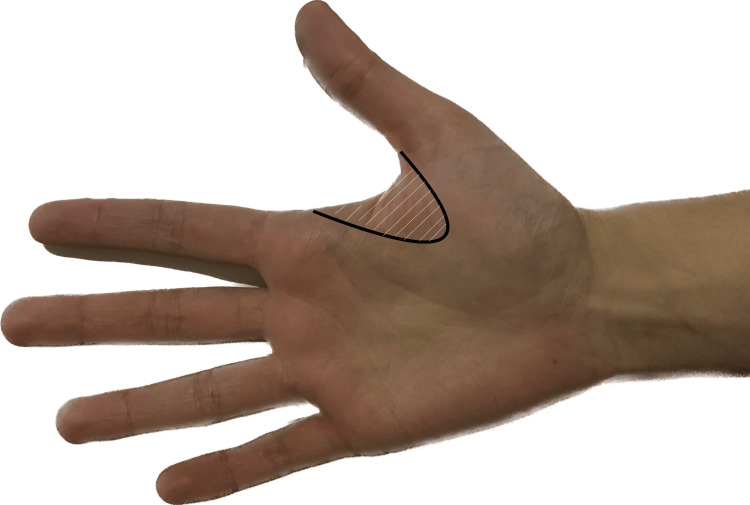
Painful area after treatment with capsaicin

Six months later, the patient reported no allodynia, with mild pain (VAS 3) in a small area in the thenar eminence (Figure 2). The hyperalgesia was replaced by hypoesthesia. The hand edema and flush had significantly reduced, and functional progress was considerable, allowing her to resume all the usual hand activities, including working as a cleaning lady. A concomitant psychological improvement was observed.

Four years after the trauma, the pain and the other dystrophy changes can be controlled with only gabapentin. She doesn’t need opioids anymore and has stopped the use of antidepressants.

## Discussion

Capsaicin acts on the transient receptor potential cation channel vanilloid type 1 (TRPV1), which modulates nociceptive inputs to the spinal cord and brain stem centers, as well as integrating diverse painful stimuli. Once applied to the skin, high concentrations of capsaicin cause a brief initial sensitization, followed by long-term desensitization mediated by the ablation of TRPV1 expressing afferent terminals, resulting in long-lasting analgesia [3].

Although capsaicin is a well-accepted and established treatment in some neuropathic pain, its use in CRPS is still controversial. In fact, even if CRPS has some neuropathic characteristics, other physiopathological mechanisms are involved. Generally, painful stimuli are not recommended for CRPS treatment, and capsaicin initially induces a strong nociceptive stimulation, which may increase the central sensitization characteristic of that pathology.

Studies on the use of capsaicin in CRPS treatment are scarce, with only a few case reports published, most of them in the context of CRPS type I [4,5]. One observational retrospective study describes a series of 120 patients, 12 with CPRS type I/II, in which capsaicin appears to reduce pain severity and painful area, but with less efficacy than in the rest of the sample; moreover, no distinction is made between type I and type II CRPS [6].

We have reported the successful use of capsaicin in a case of CRPS type II, whose application resulted in a considerable functional improvement for the patient. Our case reinforces that topical capsaicin, alone or in combination with other therapeutic strategies, can be a viable option in patients with CRPS type II, whose management can be highly complex. Additionally, the use of topical analgesia contributes to the reduction of the systemic side effects associated with other pharmacological agents. 

## Conclusions

CRPS can occur after surgery, especially if there is a history of trauma. Its early identification or suspicion and adequate treatment are important. This case report reinforces that capsaicin may play a role in the highly complex type II CRPS treatment, especially in CRPS refractory to conventional treatments. However, due to the existence of only a small number of case reports, more studies are needed on this topic.
